# Self-powered portable melt electrospinning for in situ wound dressing

**DOI:** 10.1186/s12951-020-00671-w

**Published:** 2020-08-10

**Authors:** Ying-Tao Zhao, Jun Zhang, Yuan Gao, Xiao-Fei Liu, Jiang-Jun Liu, Xiao-Xiong Wang, Hong-Fei Xiang, Yun-Ze Long

**Affiliations:** 1 Collaborative Innovation Center for Nanomaterials & Devices, College of Physics, Qingdao University, NO. 308 Ningxia Road, Qingdao, 266071 People’s Republic of China; 2 Affiliated Hospital of Qingdao University, Qingdao, 266071 People’s Republic of China

**Keywords:** Wound dressing, Electrospinning, Nanofibers, Hand-hold e-spinning gun

## Abstract

**Background:**

Electrospun (e-spun) nanofibers for wound dressing have attracted wide attention due to its large specific surface area, large porosity and breathability. Compared with solution electrospinning (e-spinning), melt e-spinning is more bio-friendly without toxic solvent participation, which provides the possibility of in situ e-spinning on wounds directly. However, previously reported melt e-spinning devices were usually bulky and cumbersome due to their necessary heating unit, and different components were separated to avoid electrostatic interference.

**Results:**

In this article, we report on a self-powered hand-held melt e-spinning gun which can work without any external power supply (outdoors). The problem of electrostatic interference for this integrated device was solved by using a special high heat transfer insulation unit. The apparatus is easy and safe to operate by a single hand due to its small volume (24 × 6 × 13 cm^3^) and light weight (about 450 g). Some biodegradable polymers, for example, polycaprolactone (PCL) fibers were successful e-spun onto wounds directly by using this dressing gun.

**Conclusions:**

PCL fibrous membrane has good biocompatibility and can be in situ electrospun to wound surface as a wound dressing by the portable melt e-spinning gun. Besides wound dressing, this hand-held melt e-spinning gun may be used in 3D printing and experimental teaching demonstration aids.

## Background

Every year, more than 6 million patients suffer from severe burns and over 300,000 people ultimately die from these injuries worldwide [1, 2]. Wound dressings play a major role in wound healing process, because it can increase wound healing and effectively prevent wound infection [3, 4]. Electrospinning (e-spinning) nanofibers for wound dressing have attracted wide attention due to its unique advantages [1, 5]. The fiber membranes have high porosity with excellent pore-interconnectivity, which is particularly important for exuding fluid from the wound and breathe freely; the inherent small pores and high specific surface area enable them to inhibit the invasion of exogenous microorganisms and assist the control of fluid drainage; the three-dimensional network scaffold structure of the fibers is similar to the natural extracellular matrix, which can provide a similar cell growth microenvironment and is more conducive to wound healing and growth [6–8]. However, most of the current dressing nanofibers are electrospun (e-spun) by solution e-spinning firstly, and then applied to the wound. Biocompatible materials, such as polycaprolactone (PCL) [9], poly(lactic acid) (PLA), poly(lactic-*co*-glycolic acid) (PLGA), are usually dissolved in toxic solvents such as acetone and chloroform, and then both of them as a spinning precursor solution for solution e-spinning, which limits the possibility of in situ spinning onto the wound [10–12]. In fact, in situ e-spinning refers to e-spinning polymer nanofibers onto the wound directly, which has a better fit than the traditional method of in vitro e-spinning and re-attachment, especially for the uneven surface of the wound [13–15]. And, there has been some work on in situ solution e-spun fibers as wound dressings [13], but there is no work on melt e-spun fibers as wound dressings because of the complexity of devices and electromagnetic interference. Besides, unlike solution e-spinning, melt e-spinning is a green and safe way for wound dressings because there is no solvent involved in the e-spinning process, so that it has no harmful solvent residues and can be directly deposited onto the human skin and organs [16–18]. Moreover, melt e-spinning can also fabricate more biocompatible polymers which have no suitable solvent at room temperature for wound dressings [19]. Therefore, these unique properties of melt e-spinning make in situ melt e-spinning more suitable for wound dressings.

In order to achieve in situ melt e-spinning, the conventional melt e-spinning device is an option [20–31]. However, these conventional melt e-spinning devices usually use a heating method such as resistance wires [22, 23], laser heating [24, 25], microwave heating [26], heated air and oil liquid [26–31] which need mains supply to melt the polymer for subsequent e-spinning, which is inconvenient in practical clinical and practical applications, especially where is no mains supply in the wild outdoors. In order to solve the problem of relying on the mains supply, the previous research used heating methods such as alcohol lamps, candles, etc. [29, 32]. However, the devices of these methods are bulky, unstable in heating, and unfavorable for portability [33]. Interestingly, previous studies have not integrated both resistance wire heating and high voltage into a device because of the strong electromagnetic interference problems that have plagued portable melt e-spinning [34, 35]. Our group has tried electric heating wire and high voltage for in situ melt e-spinning, but this device is not integrated due to electrostatic interference problems, which is not conducive to portability [21].

**Fig. 1:**
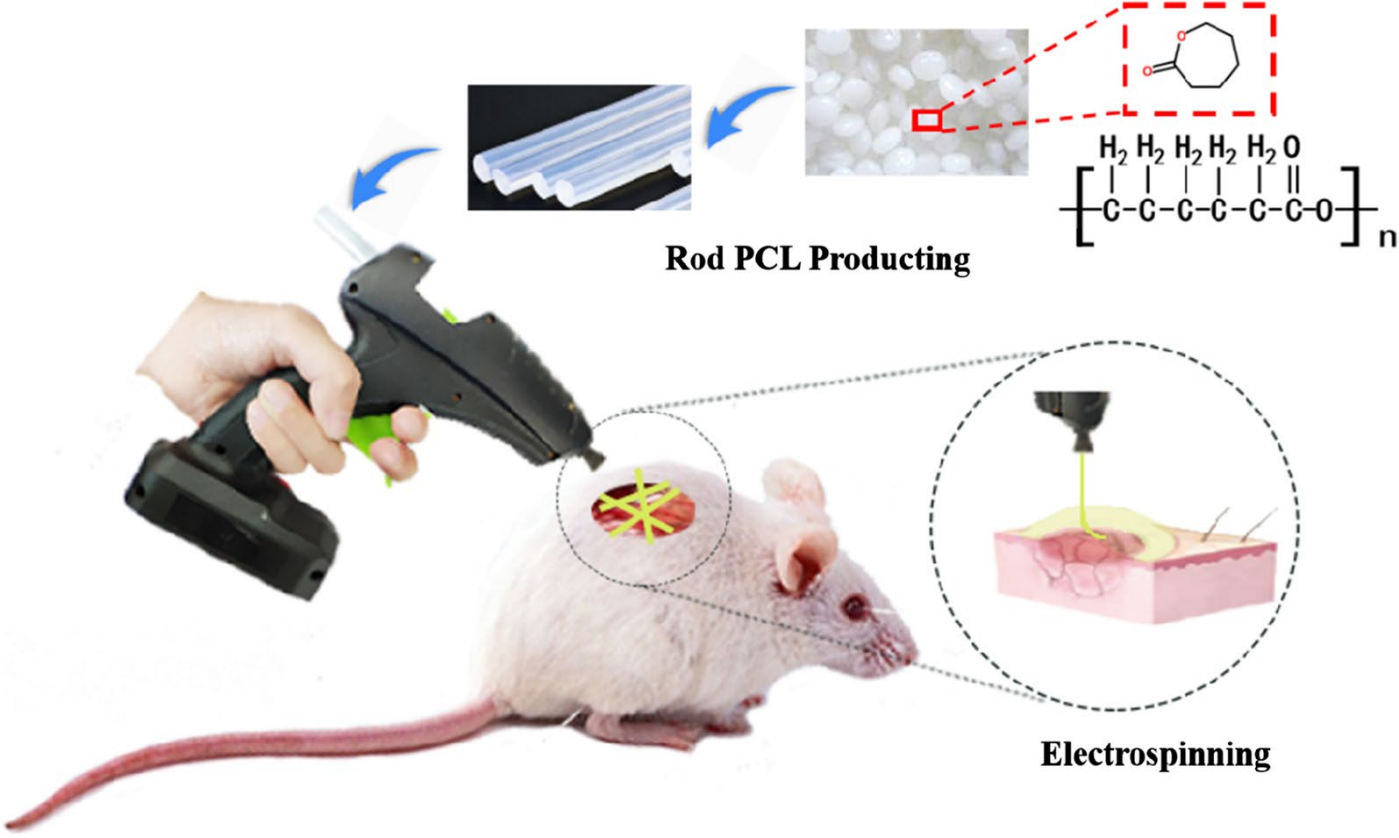
Schematic diagram of preparation of polymer nanofibers for wound dressing by in situ melt e-spinning using homemade hand-held melt e-spinning apparatus

In this study, we have designed and developed a portable in situ melt e-spinning device that only uses AAA dry batteries for self-driving, and we have also proposed a heat preservation antistatic interference unit with good thermal conductivity for the first time to overcome electromagnetic interference, making the entire device compact and portable. The whole apparatus has a light weight (about 450 g), small volume (24 × 6 × 13 cm^3^). It enables in situ melt e-spinning of a variety of polymers in the field. Moreover, melt e-spinning dressing gun enables fast e-spinning of PCL fibers onto wound directly (Fig. 1). This melt e-spinning gun may be used as personal healthcare equipment, medical equipment, cosmetic tools and experimental teaching demonstration aids.

## Methods

### Materials

Poly(lactic acid) (PLA, average molecular weight of 200,000, Nature Works), poly(lactic-*co*-glycolic acid) (PLGA 75:25, average molecular weight of 150,000, Jinan Daigang Biomaterial Co., Ltd, China), polycaprolactone (PCL, average molecular weight of 80,000, SOLVAY), and hot-melt adhesive (Linyi Zhongxin Adhesive Products Factory, China) were purchased and used without other treatments. Male SD rats (5 weeks old) were purchased from Jinan Pengyue Experimental Unit Reproduction Co., Ltd. (Qingdao, China).

### Melt e-spinning apparatus and melt e-spinning process

The self-powered hand-held melt e-spinning apparatus has a weight of about 450 g and a precise size of 24 cm in length, 6 cm in thickness and 13 cm in height, which is suitable for one-handed operation. The device comprises a heating unit, a high voltage unit, a propulsion unit and a thermal transmission electrical insulation unit. The entire apparatus does not require any external power supply and the built-in power supply allows the apparatus to operate continuously for more than 3 h. The experimental flow chart is shown in Fig. 1. First, the extruder made the PCL particles into a PCL round bar with a diameter of 11 mm and a length of 20 cm. Next, PCL round bar was placed in the device. After turning on the heating switch and 3 min preheating, the device could work at different spinning distances and using different types of needles, and the e-spun polymer fibers were deposited on the collector.

### Cell culture

Human oral fibroblasts were used for the cell experiments. The growth medium consisted of alpha modified eagle medium, 10% (v/v) fetal bovine serum, and 1% pen/strep. Cells were incubated at 37 °C with 5% CO_2_, and harvested at approximately 80–90% confluence from T25 culture flasks by trypsin of 3 min for further subcultures. All substrates with diameter of 1.5 cm were treated with UV irradiation and 70% ethanol for sterilization, placed in 24-well plates and washed by PBS. Afterward, human fibroblasts were seeded onto the samples in well plates at a density of 1 × 10^4^ cells/well. All substrates were stored in an incubator at 37 °C with 5% CO_2._

### Cytotoxicity assay

Cellular metabolic activity was measured using an CCK-8 assay (Absin). The sample membranes were cut into a circle having a diameter of 2 cm, and sterilized by UV light irradiation in a clean bench, and each side was irradiated for 30 min. The cell climbing slices were immersed in 75% alcohol for 5 min, after which the sterilized membranes were wrapped on the cell climbing slices and placed in a 24 well-plate. After adding 1 mL of cell culture medium to each well, 500 μL of 60,000 cell mL^−1^ of fibroblasts were inoculated and then incubated in a cell culture incubator for 1 day. Then, one part of the samples were taken for cell counting kit-8 (CCK-8) experiment: first removed the medium, added 400 μL of new medium and 40 μL of CCK-8 solution and gently shook the plate. Incubated the cell for 1 h in the incubator, then took it out and gently shook the plate, 150 μL of the incubated solution was added to each well of a 96-well plate. Added 200 μL of fresh medium to the 96-well plate to be the control group. Finally, detected the absorbance at 450 nm by a enzyme-labeled instrument.

### Cell staining

The remaining samples were stained to observe cell morphology: Removed the cell culture medium and rinsed it with phosphate buffer saline (PBS). Then added 4% paraformaldehyde (PFA) solution to fix the cells for 20 min. Next, rinsed three times with PBS and finally stored in PBS at a constant temperature of 4 °C. The cells were stained in the next step: Firstly, the cells were permeabilized by 0.5–1.0% Trito-x-100/PBS, removed Trito-x-100/PBS after 3 min and washed twice with PBS. Secondly, added 5% BSA/PBS solution for 30 min. Thirdly, the cytoskeleton was stained by phalloidin, after 30 min, rinsed with 1% BSA/PBS and PBS. Finally, the cell nucleus were stained with 4′,6-diamidino-2-phenylindole (DAPI), after 10 min, washed with BSA/PBS and PBS solution. Observation was performed using an inverted fluorescence microscope.

### Wound dressing experiment

Male SD rats (5 weeks old) were purchased from Jinan Pengyue Experimental Unit Reproduction Co., Ltd. (Qingdao, China). All animals were treated in accordance with the guidelines of the Chinese Government Ministry of Health Animal Laboratory Supervision and Management Committee. Anesthesia was performed with 7% chloralhydrate. First, the back of the mouse was shaved and disinfected, and then a wound of 2 cm in length was cut with a sterile surgical scalpel, followed by blood wiping and alcohol disinfection of the wound. And then e-spinning the PCL fiber with hand-held melt e-spinning apparatus. The specific operation process is shown in Fig. 7. The wound was covered with the fiber and there was no blood spill.

### Characterization

The morphology and the diameter of the melt e-spun fibers were characterized by a scanning electron microscope (SEM, TM-100, Hitachi). The spinning process was recorded by a digital video camera (EX-ZR400, Canon). PCL fibers were also examined by the Fourier transform infrared spectroscopy (FTIR, Thermo Scientific Nicolet In10) and thermogravimetric analyzer (TGA; Mettler-Toledo TGA/DSC). The OD of cell suspension was measured by a microplate reader (BioTek). The strain–stress curves of the fibers were obtained by a dynamical mechanical analyzer (Q-800, TA Scientific).

## Results and discussion

### Design of the self-powered hand-held melt e-spinning dressing gun

**Fig. 2:**
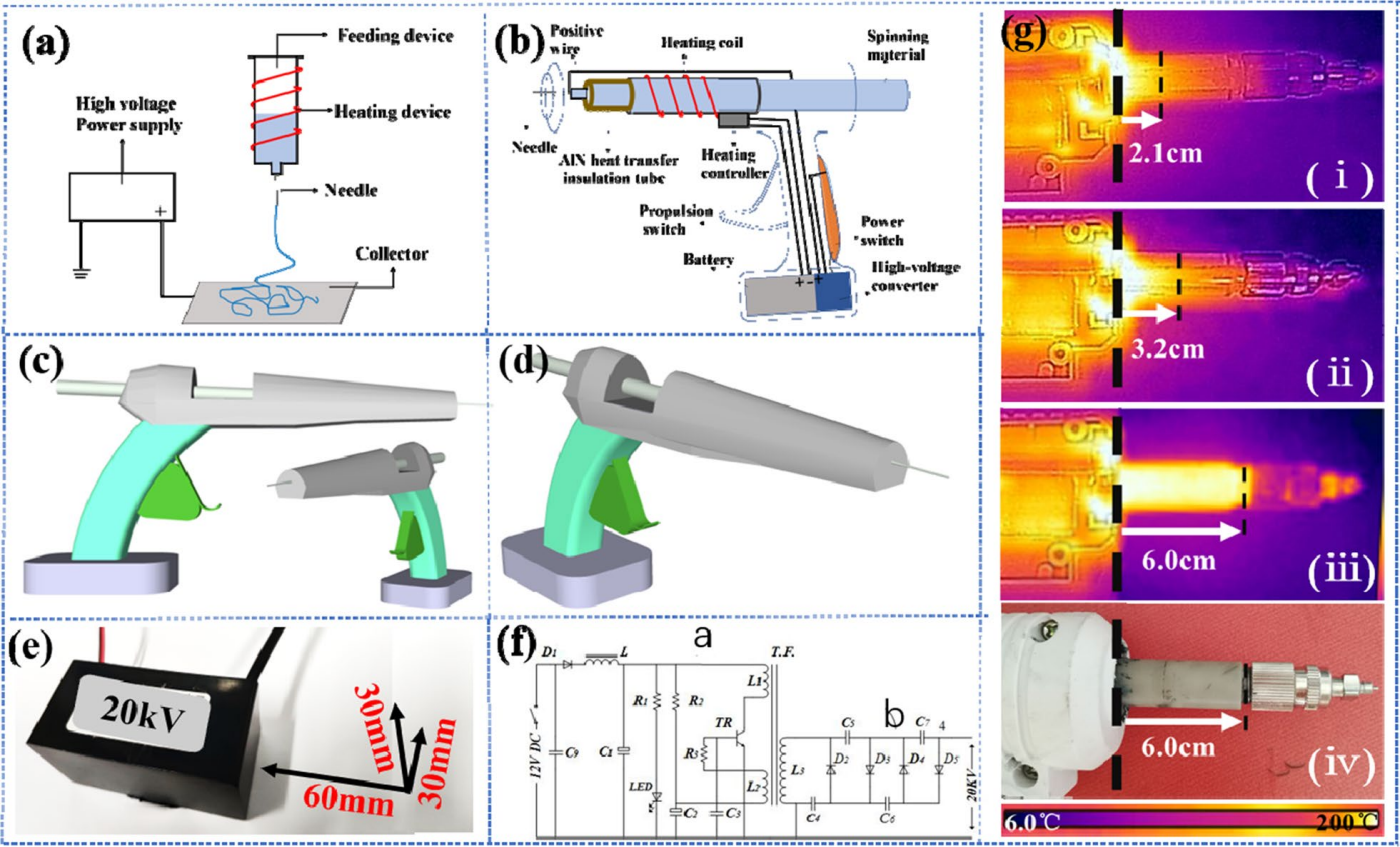
Images of homemade hand-held melt e-spinning dressing gun. The schematic diagrams of **a** a traditional melt e-spinning setup using a high voltage–power supply (HVPS) connected on collector (cannot directly receive e-spinning fibers with organisms) and **b** a battery-operated homemade hand-held melt e-spinning dressing gun which can operate outdoors and directly receive e-spinning fibers with organisms. **c**, **d** 3D model diagram of the homemade hand-held melt e-spinning dressing gun. High voltage conversion unit: **e** high voltage converter physical map with length label and **f** high voltage converter circuit schematic. **g** High heat transfer insulation unit: (**i**) Quartz tube; (**ii**) CaO tube; (**iii**) AlN tube; (**iv**) Physical map of AlN tube

As shown in Fig. 2a, traditional e-spinning device has four components: a high voltage–power supply, a heating device, a feeding device and a collection device. The volume of the traditional device is relatively large and the parts of the device are separated, so the device is not portable. Besides, the typical high voltage–power supply (HVPS) and heating unit for e-spinning requires a 220 V working voltage. As shown in Fig. 2b, self-powered hand-held melt e-spinning gun has many creative modifications. Firstly, the size of the entire device has been reduced several times and the device is more integrated, as shown in Fig. 2c, d. The self-powered hand-held melt e-spinning gun has a weight of about 450 g and a precise size of 24 cm in length, 6 cm in thickness and 13 cm in height, which is realize the portability of the device. Secondly, the heating unit is fully powered by a rechargeable battery. Heater strip and thermal controller are directly connected to the 12 V battery by wire. The heating wire is tightly wound around the surface of the pipe, so that the pipe can quickly increase the temperature to melt the polymer. Then, the melted polymer is extruded by a pressing device for e-spinning. Thirdly, a high-voltage converter and the rechargeable lithium battery are employed to provide a high voltage instead of the typical HVPS of 220 V working voltage. As shown in Fig. 2b, the battery provides a 12 V voltage and connected to the converter. The high-voltage converter physical map and circuit schematic are showed in Fig. 2e, f. The high-voltage converter includes an oscillating circuit and a voltage doubling circuit. After the power supplied, the oscillating circuit generates high-frequency oscillation, and the high-voltage oscillating current is induced by the high-voltage induction coil L_3_, and then boosted to a high voltage of 20 kV by the voltage doubling circuit. The high voltage generated by the high-voltage converter is connected to the spinning needle by a positive wire. The negative electrode of the converter is connected to a metal piece on the handle. In this way, the charge can be transferred through the body (hand) touching the metal piece to avoid charge accumulation. Finally, the device is equipped with a high heat transfer electrical insulation unit to fulfill the heat transfer and electrostatic isolation. This unite has a high material requirement and was first proposed in this article. Materials with high thermal conductivity, insulation and high plasticity are rare. The homemade threaded AlN tube has good electrical insulation and heat transfer capacity. As shown in Fig. 2g, compared to CaO ceramic tubes and quartz tubes, AlN tube exhibits ultra-high heat transfer capacity. In addition, the surface of the AlN tube is covered with a layer of thermal insulation cotton to reduce heat loss. It has been proved by experiments that a 6 cm AlN tube covered with a layer of thermal insulation cotton is the most suitable and safest as the high heat transfer insulation unit.

All in all, the integrated apparatus is easy to handle and operate safely. The entire apparatus does not require any external power supply and the built-in power supply allows the apparatus to operate continuously for more than 2 h. Therefore, we can spin fiber membrane using the hand-held melt e-spinning apparatus anywhere and anytime when needed. In addition, its working life can be prolonged by charging the lithium battery when the device is idle.

### Performance of the self-powered hand-held melt e-spinning dressing gun

**Fig. 3:**
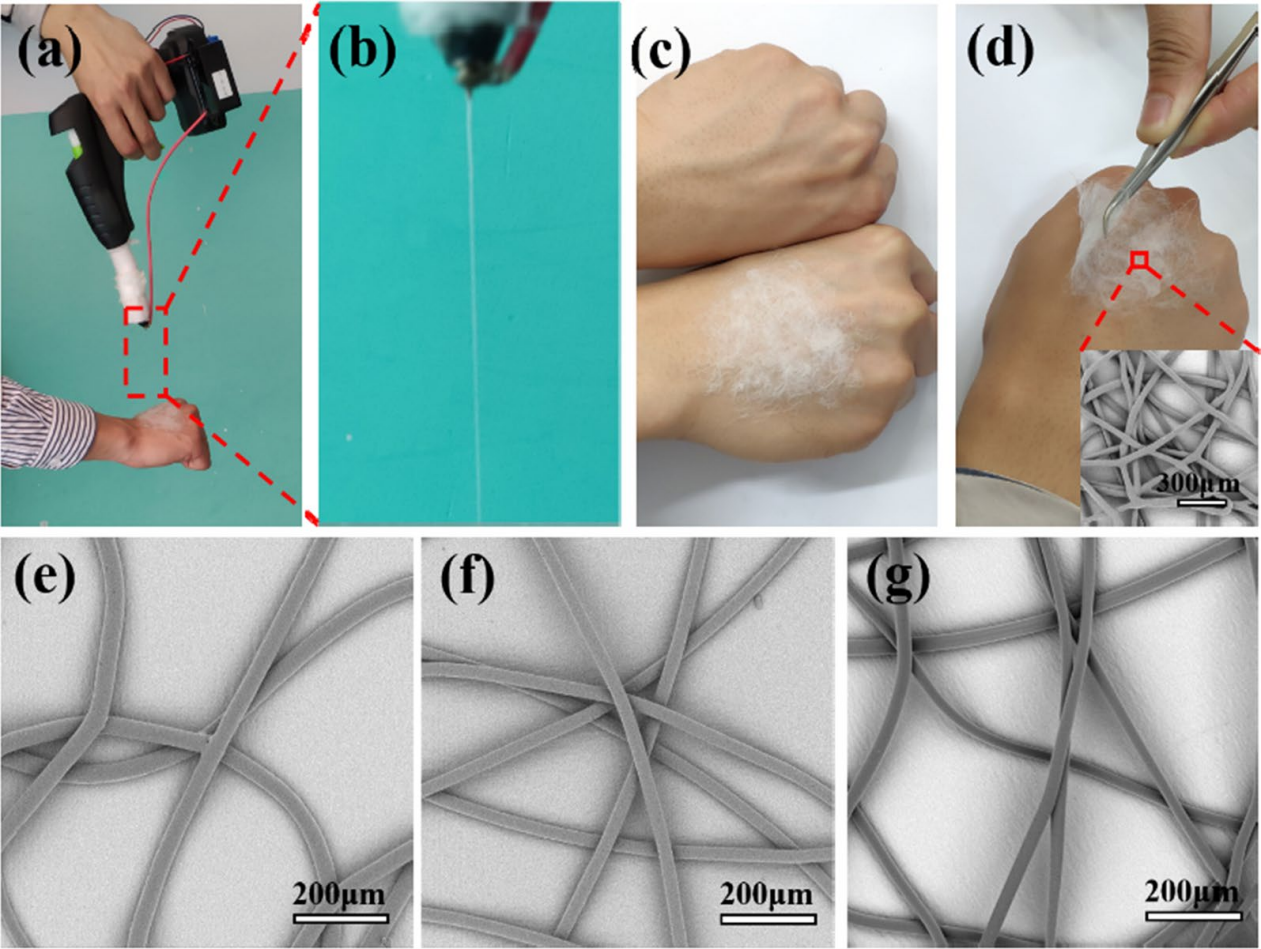
Optical pictures showing the process of melt e-spinning PCL fibers directly onto the skin producing by the hand-held melt e-spinning apparatus in 5 min and SEM images of fibers with various polymer materials produced by the hand-held melt e-spinning apparatus to further test the performances of the apparatus. **a** The apparatus was operated by one hand and the other hand receives the PCL fibers. **b** Magnified view of spinning jet. **c** Comparison of two hands with or without fiber membrane. **d** The picture shows the e-spun fiber membrane has good flexibility and the inset SEM picture is the e-spun fibers. **e** PLA fibers. **f** PLGA fibers. **g** hot-melt adhesive fibers

Figure 3a–d shows the process of melt e-spinning PCL fibers directly onto the skin producing by the hand-held melt e-spinning apparatus in 5 min. As shown in Fig. 3a, the apparatus was operated by one hand and the other hand received the PCL fibers. Partial enlargement of the spinning jet is shown in Fig. 3b. Its spinning process and principle are very similar to the traditional melt e-spinning devices. The polymer is heated to a molten state, and then overcomes the viscous resistance between the polymer molecules under the action of an external electric field; the melt jet stretches and solidifies into a fiber as the melt cools [33]. In addition, the device has better production efficiency, which can be demonstrated by the PCL fiber membrane (Fig. 3c). As shown in Fig. 3d, the PCL fiber membrane has good flexibility. Furthermore, we can see that the e-spun PCL fibers are smooth and uniform from the inset in Fig. 3d. The apparatus exhibits a good e-spinning ability.

Furthermore, in order to verify the universal applicability of the e-spinning gun, multiple polymer materials including PLA, PLGA, and hot-melt adhesive have been successfully e-spun into fibers by using the designed hand-held melt e-spinning apparatus. These polymers have good biocompatibility and have a wide range of applications. Figure 3e–g shows the SEM images of the fibers prepared by the designed device and the spinning distance is 10 cm. Besides, through diameter analysis, the prepared PLA, PLGA, and hot-melt adhesive fibers have an average diameter of approximately 27.5 μm, 21.2 μm and 35.8 μm. It is observed that the fibers are very smoothly. These experimental results demonstrate the feasibility and stability of the designed apparatus. And Additional file 1: Figure S1 shows the better fit of in situ e-spinning fibers than the traditional method, illustrating the potential application of the device in the field of wound dressings.

### The influence of e-spinning parameters on fibers

**Fig. 4:**
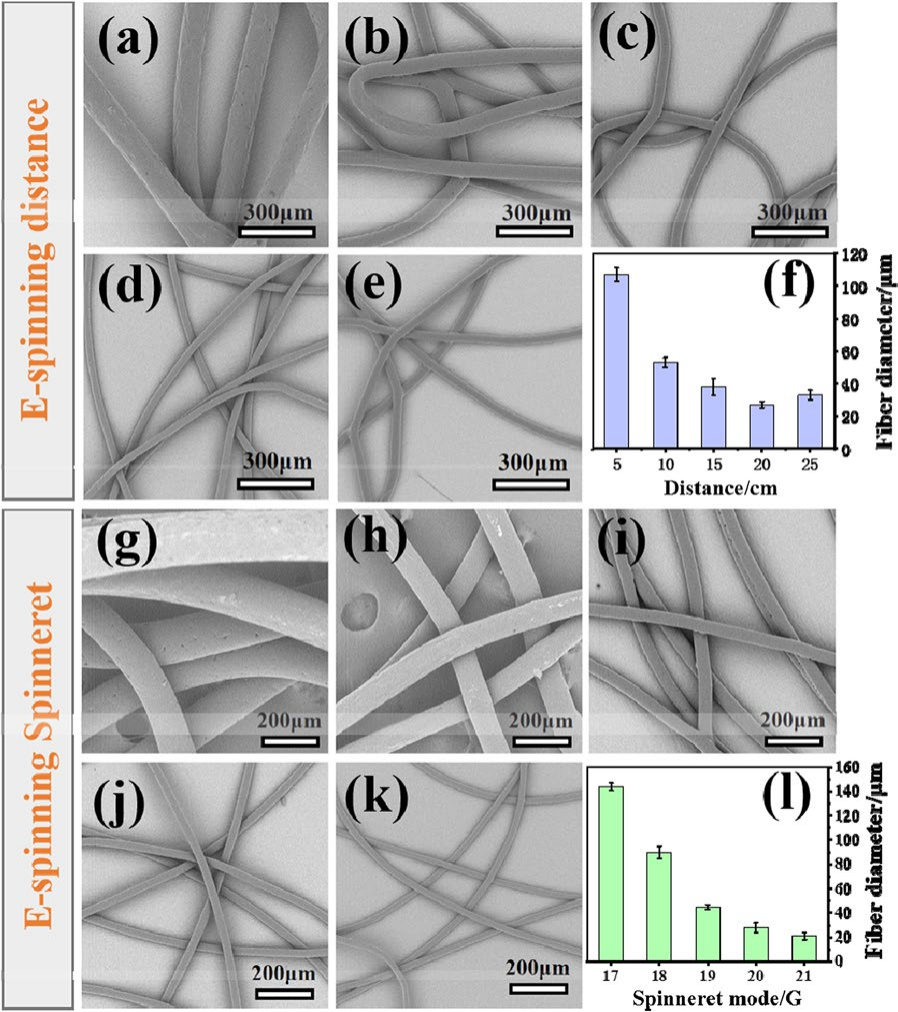
SEM images of the PCL fibers e-spun by using the hand-held apparatus under different spinning distances of **a** 5 cm, **b** 10 cm, **c** 15 cm, **d** 20 cm, **e** 25 cm. **f** statistical histogram of average fiber diameters. SEM images of the PCL fibers e-spun by using the hand-held apparatus under a spinning distance of 15 cm within different inner diameter spinneret. The spinneret model of **g** 17 G (1.07 mm), **h** 18 G (0.86 mm), **i** 19 G (0.67 mm), **j** 20 G (0.60 mm), **k** 21 G (0.50 mm). **l** Statistical histogram of average fiber diameters

For portability, the apparatus’ high voltage generator consists of a 12 V rechargeable lithium battery and a high-voltage converter. Although the voltage is fixed, during the melt e-spinning process, the electric field strength is controlled by adjusting the distance from the needle to the collector. Besides the distance from the needle to the collector directly affects the degree of stretching and flight time of the spinning jet in the electric field. Therefore, e-spinning distance is a very important parameter affecting fiber morphology and we investigated the effect of e-spinning distance on e-spun fibers diameter. Figure 4a–e show the SEM images of the PCL prepared in different e-spinning distance in the range of 5 to 25 cm. it is found that the PCL fibers average diameter reduced from 103.2 μm to 27.4 μm when the e-spinning distance increased from 5 to 20 cm and when the spinning distance is increased to 25 cm the fiber diameter increased to 37.2 μm. This is because the spinning distance has a double effect on the fiber diameter. A larger receiving distance provides sufficient time for the jet to be sufficiently stretched to reduce the diameter of the fiber; on the other hand, large fiber receiving distance reduces the electric field strength and makes the stretching of fibers weak, resulting in an increase in fiber diameter [21, 29]. The optical morphology picture of fiber membrane deposited on a pork liver is showing in Additional file 1: Figure S2b. Furthermore, the type of spinning needle is another important factor affecting the fiber diameter. The SEM images of the as-spun PCL fibers prepared under different spinning needle in the range of 17–21 G were shown in Fig. 4g–k. The fiber diameter is decreasing as the spinning needle becomes thinner, which is agrees with the former study [32]. Therefore, we can change different types of spinning needles according to different needs, which is convenient and quick.

### FT-IR and TGA of the e-spun PCL fiber dressing

**Fig. 5:**
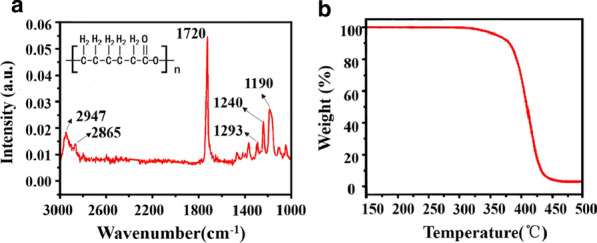
**a** FTIR spectra and **b** TGA spectra of the melt e-spun PCL fibers by using the hand-held apparatus

The FTIR spectra obtained from PCL fiber dressing prepared by the designed apparatus, are shown in Fig. 5a. The chemical structure of PCL are obviously found from the FTIR spectra, such as the stretching vibration peak at 2867 cm^−1^ and 2947 cm^−1^ belong to C–H bond of CH_2_ group, and The absorption band that appeared at 1730 cm^−1^ was assigned to the C=C bond, and asymmetric C–O–C, C–C, OC–O stretching in FTIR located at 1244 cm^−1^, 1295 cm^−1^, 1191 cm^−1^, respectively. The FTIR spectroscopy of the e-spun fibers indicates that the designed melt device fabricates fibers without breaking chemical bonds that often can be from the decomposition of polymers. [36, 37] In the TGA, the PCL degradation profile showed high thermal stability, which started to degrade at 327 °C and completely decomposed (0% w/w char residue) at 476 °C. [38] The weight loss zone of PCL is 327–476 °C. Therefore, 200 °C of the melt spinning temperature does not cause PCL to be decomposed, which is consistent with FTIR results. Besides, the stress–strain curve of the fiber was also tested to show the good stretchability (Additional file 1: Figure S2).

### Biocompatibility experiment

**Fig. 6:**
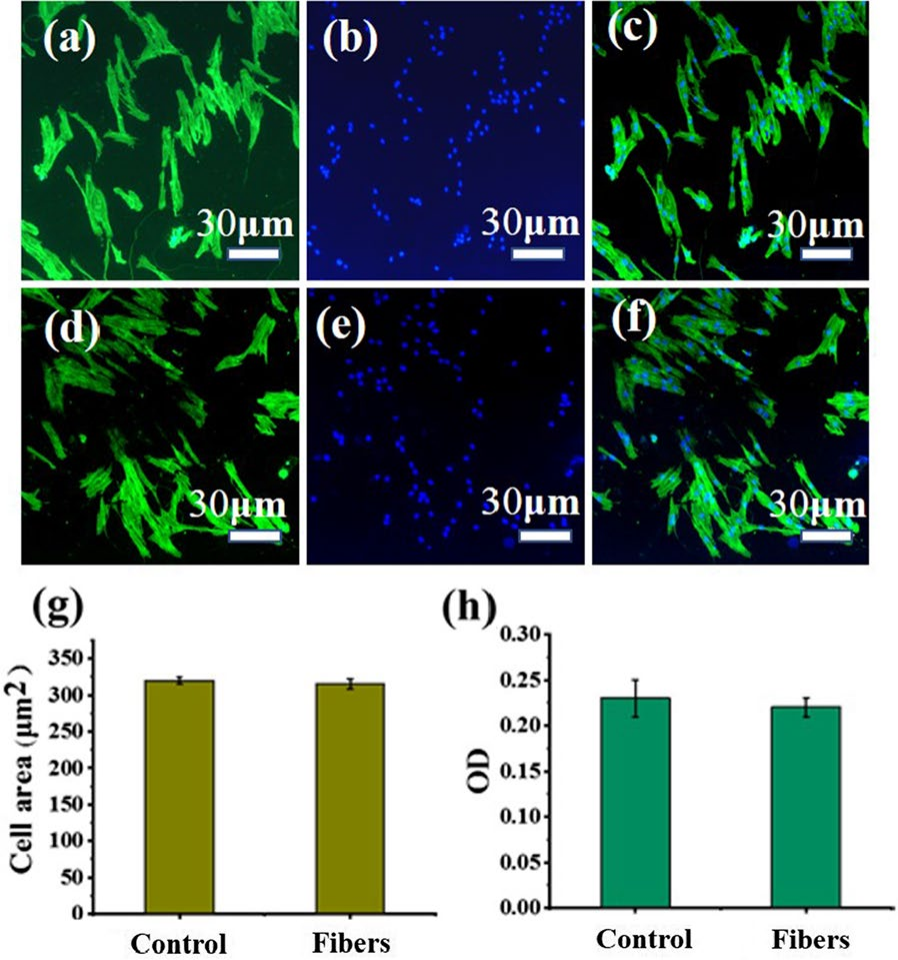
Cytoskeleton analysis of fibroblasts on the **a**–**c** the melt e-spun PCL fiber membrane and **d**–**f** TCP stained with phalloidin and DAPI after one day incubation. **g** Average cell area and **h** OD value of fibroblasts on these two different substrates

For evaluating the effect of nanofibers on cells, biocompatibility assays were performed. Tissue culture polystyrene (TCPs) has been used to culture adherent cells of human oral fibroblasts, and was used as a control culture system in this research. Because cells could be adhered normally and healthy by culturing cells on TCPs, and cellular behaviors can be compared between two-dimensional TCPs and three-dimensional nanofiber mats. [39, 40] We seeded human oral fibroblasts on substrates for 1 day. The attachment and morphology of human fibroblasts were determined with a double-label fluorescence staining of actin cytoskeleton and nucleus. As shown in the Fig. 6, the cells on the fiber membrane and TCPs both showed a similar spindle morphology. By comparing the average area of the fibroblasts in the two groups, it can be seen that the area of the cells on the membrane and TCP was almost the same. This is because that nanofibers supply a topographic environment to which cells attach well by local cytomembrane wrapping [40]. The result can further explain that the e-spun membrane does not adversely affect the growth of the cells (Fig. 6c).

Besides, CCK-8 assays were performed to evaluate the viability of human fibroblasts on the melt e-spun fibrous membranes. OD values of TCPs and fibers are similar. This result is consistent with some previous literature reports. Shin et al. put forward that cell adhesion was higher on PCL nanofibers than on TCPs [40]. And in their experiments, OD values of TCPs and fibers are similar. Nanofibers supply a topographic environment to which cells attach well by local cytomembrane wrapping. [40] And e-spun PCL fibers, creating a pore size of micron order, provides the good microenvironmental conditions for attached/proliferated cells. [41, 42] There is not significant difference between two different substrates, which indicates that there were no cytotoxic effects of the melt e-spun fibrous membranes. Cytocompatibility experiments demonstrate that fiber membranes can be used as a cell-permissible material for more medical attempts.

### Wound dressing experiment

**Fig. 7:**
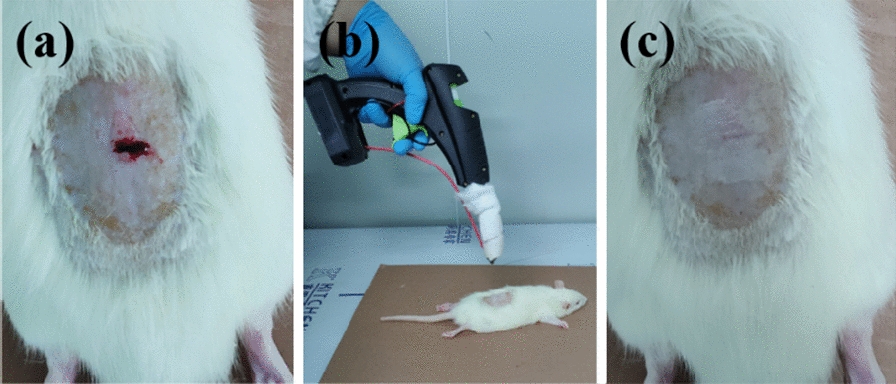
Diagrams and optical images of melt e-spinning PCL fibers onto the wound of a rat by using the hand-held apparatus. **a** A wound of about 1 cm was made on the back skin of the rat and the blood flowed out. **b** The process of PCL fibers e-spun on the wound after cleaned and disinfected by gauze soaked in alcohol. **c** The wound was stuck by the fiber and there was no blood spill

Compared with powder spray and gauze, the fiber membranes have high porosity with excellent pore-interconnectivity, which is particularly important for exuding fluid from the wound and breathe freely; the inherent small pores and high specific surface area enable them to inhibit the invasion of exogenous microorganisms and assist the control of fluid drainage; the 3D network scaffold structure of the fibers is similar to the natural extracellular matrix, which can provide a similar cell growth microenvironment and is more conducive to wound healing and growth [6–8]. PCL, a semi-crystalline polyester that is biodegradable and biocompatible, has been widely investigated in wound dressings and tissue engineering [43]. Moreover, although the fibers are micro-sized, there are some research prove that microfibers also have good absorbance values in MTT test and induce good tissue infiltration [44]. Therefore, microfibers are also often applied in wound dressing and tissue engineering. [44–46] And the apparatus we designed perfectly matched to the preparation of nanofiber membrane. Therefore, we conducted an experimental exploration and the process is shown in Fig. 7. First, we selected a test mouse and cut a wound on its back, followed by blood wiping and alcohol disinfection of the wound, and then e-spinning the PCL fiber with hand-held melt e-spinning apparatus. The specific operation process is shown in Fig. 7. The wound was stuck by the fiber and there is no blood spill.

Furthermore, the temperature of the deposited fibers on the wound were measured by Infrared Thermometer under different e-spinning distances, as shown in Table 1. We can see that the temperature of the deposited fiber is close to the temperature of the living body. Therefore, the melt-spun PCL fibers are suitable for application in wound dressing.

**Table 1:** Measurement results of the deposited fibers’ temperature

Material	Spinning distance (cm)	Temperature of mouse skin before e-spinning (°C)	Temperature of material barrel (°C)	Temperature of spinneret (°C)	Temperature of dropped fibers on skin (°C)
PCL fibers	7	36–38	180–200	124–130	38.5
	10	36–38	180–200	124–130	34.7
	13	36–38	180–200	124–130	30.2

Other than this, functional wound dressing can be prepared using this system by adding materials with antibacterial, hemostatic and other substances into PCL, such as: antibacterial Ag nanoparticles, then the composite wound dressings have antibacterial properties. Therefore, we can prepare multifunctional wound dressing by self-powered hand-held melt e-spinning apparatus conveniently.

## Conclusions

In this work, we present a self-powered hand-held melt e-spinning gun using a rechargeable battery to provide a high voltage and heating power, which can work without any extra electricity supply. The high thermal transmission electrical insulation unit was first proposed and successfully solved the electrostatic interference between the needle with high-voltage and the heating unit. The whole apparatus has small volume, light weight and can be operated by a hand. The operation of the apparatus is easier and safer. Various materials (PCL, PLA and PLGA) were successfully melt e-spun into the smooth fibers and the e-spinning parameters such as e-spinning distance and spinneret diameter on fibers morphology were investigated. All in all, we could spin fiber membrane by using this hand-held melt e-spinning apparatus anywhere and anytime when we need. Additionally, the wound dressing was fabricated by in situ melt e-spinning fibers onto the skin directly by using the apparatus. All the characterizations reveal that the self-powered hand-held melt e-spinning gun may be useful in wound dressing and other fields.

## Supplementary information

**Additional file 1: Figure S1.** (a) In situ e-spinning fibers deposited on the skin, showing good adhesiveness. (b) Traditional fibers which are e-spun firstly and then applied to the skin, showing bad adhesiveness. **Figure S2.** (a) Stress–strain curve of the melt e-spun PCL fibers. (b) Optical picture of fiber membrane deposited on the surface of a pork liver.

## Data Availability

All data generated or analyzed during this study are included in this article.
