# Supplementary material for: Self-powered portable melt electrospinning for in situ wound dressing
Source: J Nanobiotechnology. 2020 Aug 10;18:111. doi: 10.1186/s12951-020-00671-w (PMC7416801; doi:10.1186/s12951-020-00671-w)
Supplement: Supplementary file 1 — Additional file 1: Figure S1. (a) In situ e-spinning fibers deposited on the skin, showing good adhesiveness. (b) Traditional fibers which are e-spun firstly and then applied to the skin, showing bad adhesiveness. Figure S2. (a) Stress–strain curve of the melt e-spun PCL fibers. (b) Optical picture of fiber membrane deposited on the surface of a pork liver. [file 12951_2020_671_MOESM1_ESM.doc]

Additional Information

**Self-powered portable melt electrospinning for in situ wound dressing**

*Ying-Tao Zhao^a,†^, Jun Zhang^a,†^, Yuan Gao^a^, Xiao-Fei Liu^a^, Jiang-Jun Liu^b^, Xiao-Xiong Wang^a^, Hong-Fei Xiang^b^, and Yun-Ze Long^a ,*^*

^a^ Collaborative Innovation Center for Nanomaterials & Devices, College of Physics, Qingdao University, Qingdao 266071, P. R. China

^b^ Affiliated Hospital of Qingdao University, Qingdao 266071, P. R. China

______________________

^†^These two authors contributed equally to this work.

^*^Corresponding author.

E-mail: [yunze.long@163.com](mailto:yunze.long@163.com) or [yunze.long@qdu.edu.cn](mailto:yunze.long@qdu.edu.cn)


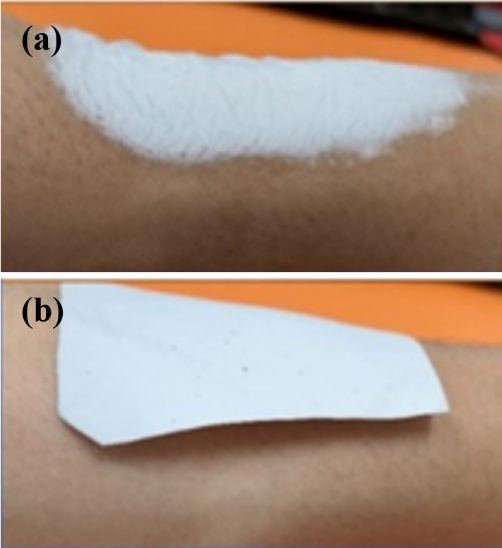


**Figure S1** (a) In situ e-spinning fibers deposited on the skin, showing good adhesiveness. (b) Traditional fibers which are e-spun firstly and then applied to the skin, showing bad adhesiveness.

We carried out the comparative experiments to show the better fit of in situ e-spinning fibers than the traditional method. As shown in Fig. S1a, due to the softness of the fibers and electrostatic adherence, the fibers fit well on the surface of the skin and are not easy to tear off. In contrast, traditional dressing fibers that are e-spun firstly and then applied onto the wound show bad fit on skin (Fig. S1b).

**
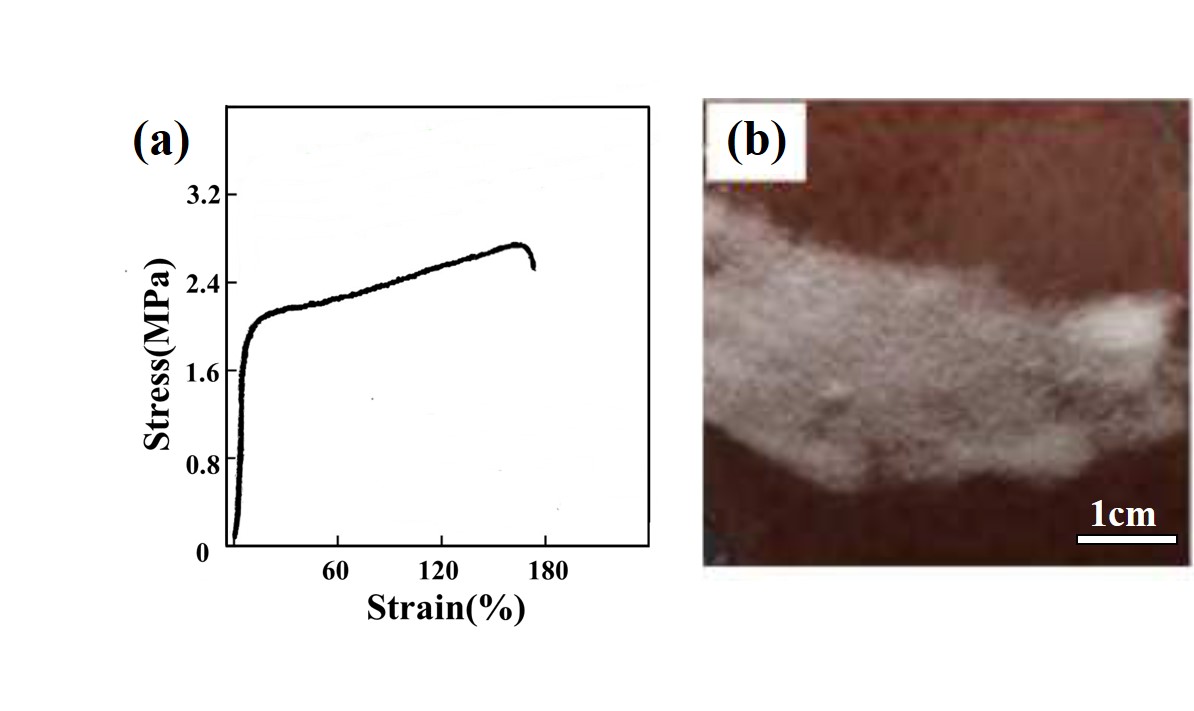
**

**Figure S2** (a) Stress-strain curve of the melt e-spun PCL fibers. (b) Optical picture of fiber membrane deposited on the surface of a pork liver.

In addition, we made the stress-strain curve of the fibers to show the good stretchability which is important for wound dressing. The stretchability of the PCL fiber was tested using a tensile tester at a fixture speed of 2 mm min^-1^ at 25 °C. As shown in the Fig. S2a, the elongation at break was up to 156% and tensile stress was 2.85 MPa, which is consistent with the previous reports and suit for wound dressing. [1]. And Fig. S2b is the optical picture of e-spun fibers deposited on the surface of a pork liver, showing a good fit.

[1] Jung S M, Yoon G H, Lee H C, et al. Chitosan nanoparticle/PCL nanofiber composite for wound dressing and drug delivery. Journal of Biomaterials Science, Polymer Edition, 2015; 26(4): 252-263.
